# CAR T Cell Therapy’s Potential for Pediatric Brain Tumors

**DOI:** 10.3390/cancers13215445

**Published:** 2021-10-29

**Authors:** Pauline Thomas, Natacha Galopin, Emma Bonérandi, Béatrice Clémenceau, Sophie Fougeray, Stéphane Birklé

**Affiliations:** 1 Université de Nantes, INSERM, CRCINA, F-44000 Nantes, France; Pauline.Thomas@univ-nantes.fr (P.T.); Natacha.Galopin@univ-nantes.fr (N.G.); emma.bonerandi@etu.univ-nantes.fr (E.B.); Sophie.Fougeray@univ-nantes.fr (S.F.); 2 Université de Nantes, CHU Nantes, CNRS, INSERM, CRCINA, F-44000 Nantes, France; Beatrice.Clemenceau@univ-nantes.fr

**Keywords:** chimeric antigen receptor, T cell, medulloblastoma, atypical teratoid rhabdoid tumors, ependymoma, high-grade glioma, pediatric brain tumor, radiotherapy, tumor microenvironment

## Abstract

**Simple Summary:**

T cells that are genetically engineered to express chimeric antigen receptors constitute an effective new therapy with curative potential for patients with hematological tumors. The value of chimeric antigen receptor T cells in childhood brain tumors, the leading cause of cancer death in children, is less clear. In this context, the main obstacles for these engineered T cells remain how to find them, allow them to infiltrate, and induce them to remain active in the tumor site. Here, we discuss recent progress in the field and examine future directions for realizing the potential of this therapy.

**Abstract:**

Malignant central nervous system tumors are the leading cause of cancer death in children. Progress in high-throughput molecular techniques has increased the molecular understanding of these tumors, but the outcomes are still poor. Even when efficacious, surgery, radiation, and chemotherapy cause neurologic and neurocognitive morbidity. Adoptive cell therapy with autologous CD19 chimeric antigen receptor T cells (CAR T) has demonstrated remarkable remission rates in patients with relapsed refractory B cell malignancies. Unfortunately, tumor heterogeneity, the identification of appropriate target antigens, and location in a growing brain behind the blood–brain barrier within a specific suppressive immune microenvironment restrict the efficacy of this strategy in pediatric neuro-oncology. In addition, the vulnerability of the brain to unrepairable tissue damage raises important safety concerns. Recent preclinical findings, however, have provided a strong rationale for clinical trials of this approach in patients. Here, we examine the most important challenges associated with the development of CAR T cell immunotherapy and further present the latest preclinical strategies intending to optimize genetically engineered T cells’ efficiency and safety in the field of pediatric neuro-oncology.

## 1. Introduction

Primary brain tumors constitute the most common solid neoplasms in children and have overtaken leukemia as the leading cause of death by cancer in childhood [1]. They constitute a group of heterogeneous tumors with different histologic, physiopathologic, and molecular features that are correlated with their prognoses [2]. The World Health Organization classifies the majority of pediatric brain tumors (PBTs) as grade 1, 2, or low-grade gliomas. Most of these tumors are curable by surgery with good clinical outcomes. However, a large proportion of PBTs are classified by the WHO as grade 3 and grade 4. Despite treatment, this group of PBTs invariably progresses rapidly with a five-year survival rate of less than 20%.

In the current WHO guidelines, PBT diagnoses and prognoses differ with WHO grade and histologic as well as molecular classification (Table 1) [2]. This combined histomolecular approach allows for a much more accurate diagnosis and, furthermore, has recently translated into novel therapeutic options for this population of patients, including targeted therapies. However, the latter have not been satisfactory in terms of patient outcomes [3]. Disease recurrence still occurs and remains lethal for children with the worst prognoses [4]. In addition, the few long-term survivors experience devastating morbidities such as cognitive and developmental disorders, in addition to a high incidence of secondary tumors [5,6]. There is, therefore, an urgent need to develop new therapies.

One promising modality is genetically modifying the patient’s T cells to express chimeric antigen receptors (CARs) that are able to bind tumor antigens in a major-histocompatibility-complex-independent fashion. Structurally, a chimeric antigen receptor has a modular design composed of (i) an extracellular antigen-binding domain, (ii) a hinge region, (iii) a transmembrane domain, and (iv) an intracellular signaling domain. The last initiates tumor cell killing, cytokine release, and antigen-dependent T cell proliferation upon antigen binding [7]. Classically, the antigen-binding domain is a single-chain fragment variable (scFv) of an antibody. Thus, any antigen recognized by an antibody can be used as a CAR target, which includes carbohydrate epitopes that cannot be recognized easily by TCRs.

For use in patients, the primary material for manufacturing CAR T cells is mononuclear cell samples collected from apheresis products. The T cell population is activated using CD3/CD28-coated immunomagnetic beads, and genetic modification is achieved using lentiviral or γ-retroviral vectors. Following genetic modification, the CAR T cells are expanded in vitro to achieve the numbers required for clinical administration [8]. To date, the most common design used is a second-generation CAR that contains either CD28 or 4-1BB costimulatory domains [9]. This CAR design has induced long-lasting complete remission in most patients with CD19-expressing B cell chemorefractory malignancies [10,11]. These unprecedented response rates have led to the marketing authorization of three CAR T cell therapies named Yescarta, Kymriah, and Brexu-cel [10,11,12]. However, life-threatening cytokine release syndrome, clinical manifestations of severe neurotoxicity, and antigen loss remain unsolved issues [13,14].

In addition to hematological malignancies, important efforts have been made to extend the potential of CAR T cell therapy to solid tumors [15]. The scarcity of suitable CAR target antigens, CAR T cell trafficking, and persistence, as well as the immune-suppressive tumor microenvironment, have presented major obstacles to the effectiveness of this strategy for the treatment of solid tumors.

Brain tumor-targeting CAR T cells offer promising perspectives for improving pediatric patient outcomes regarding their capacity to fight these infiltrative tumors. Treating brain tumors with CAR T cells remains, however, far more difficult than treating hematologic malignancies in such a manner [16]. Several important breakthroughs have been reported recently, especially with adult brain tumors, where a transient yet complete remission in a patient with disseminated glioblastoma (GBM) treated with IL13Rα2-specific CAR T cells has been reported [17]. This promising result has led to the investigation of this strategy in preclinical models of PBTs, followed by the initiation of clinical trials. While PBTs share some characteristics with adult brain tumors, they also fundamentally differ from their adult counterparts. They display several unique characteristics that hamper clinical translation, including the developing brain as a unique tumor site, the paucity of target antigens, issues of blood–brain barrier penetration, the associated tumor immunosuppressive environment, and the very small number of patients with each given brain tumor type.

Advanced CAR design engineering has enabled the further optimization of each CAR component to confer additional T cell functions beyond cytolysis to address the above hurdles, as extensively reviewed elsewhere [18,19]. Here, we review the current state of CAR T cell immunotherapies in pediatric neuro-oncology and the challenges that must be addressed in order to realize the potential of this therapy in the future. We discuss recent highlights, which provide new rationales for increasing the efficacy and safety of CAR T cell therapy in children with brain tumors.

## 2. Pediatric Brain Tumor Heterogeneity

### 2.1. Heterogeneity of Pediatric Brain Tumors

PBTs are notably distinct from adult central nervous system (CNS) tumors in terms of incidence, histology, biology, and prognosis [20,21]. To date, the established risk factors for PBTs are exposure to ionizing radiation and some rare genetic syndromes. They arise from different cell types in different locations within the CNS. About 15% of brain tumors are located in the cerebellum, and about 10% of the tumors are located in the stem [21]. They are classified into two major categories: glial and neuronal tumors. The most frequently diagnosed glial tumors are astrocytomas (WHO grade 2), oligodendrogliomas (WHO grade 2), and ependymomas (WHO grade 2 or 3). Another rare but often fatal glial tumor that occurs in the pediatric population is diffuse intrinsic pontine glioma (DIPG, WHO grade 4). The most common tumors in the embryonal group are medulloblastomas (WHO grade 4) and atypical teratoid/rhabdoid tumors (ATRTs; WHO grade 4) [22]. The frequencies of different types of PBTs differ with age. For instance, the most frequent PBTs are medulloblastomas in children aged 0–4 years. At ages 5–9 years, the most commonly diagnosed tumors are astrocytic/oligodendrional tumors, and in the age group of 10–14 years, DIPGs [23]. In addition, each group of tumors presents heterogeneity both among patients and within one tumor, as discussed further on.

Historically, CNS tumors were mainly classified according to histological characteristics. During the past decade, however, the genomic and epigenomic profiling of these tumors have evidenced specific genes and signaling pathways that are involved in their development, and this has led to the identification of multiple CNS subtypes [24]. In 2021, the WHO renewed its classification, in which the scheme for the diagnosis of PBT is based on a morphological/molecular approach that includes genetic and epigenetic tumor signatures (Table 1) [25].

PBT heterogeneity exists at several levels: (i) within an individual tumor, (ii) within an individual patient, and (iii) among the subtypes of a specific type of cancer. In fact, the tumor mass is composed of heterogeneous populations of both tumor cells and stroma cells, as discussed in Section 4. This results in a large range of variability in different biological processes, including cell metabolic profiles, cell proliferation, cell invasion, and cell sensitivity to conventional treatments. In particular, resistance to a chemoradiation therapeutic regimen has been attributed to the presence of a subpopulation of cells known as cancer stem-like cells, which reside within the tumor bulk and maintain the self-renewal and recurrence potential of the tumor [26,27]. Due to the high metabolic activity of cancer cells, the tumor microenvironment (TME) is also characterized by oxygen and nutrient competition as well as the accumulation of metabolites, which, together, often favor an immunosuppressive phenotype [28]. As such, each PBT type has specific microenvironmental characteristics affecting its own developmental process, therapeutic response, and outcome [29].

Depending on the tumor type and the age of the child, treatment options include maximal safe surgical resection combined with radiotherapy and chemotherapy [30]. Unlike many other neoplasms, the treatment of PBTs remains particularly challenging. Their location hinders surgery, and complete surgical resection is often impossible due to their infiltrative growth pattern. The presence of the blood–brain barrier (BBB) prevents a drug from reaching a clinically effective concentration at the tumor site [30]. Current therapies are otherwise associated with significant treatment-related morbidities, including endocrine dysfunctions, cognitive disorders, neuropsychologic diseases, and, in some cases, secondary tumors [31]. As such, children under three years of age cannot benefit from radiation therapy due to the devastating neurocognitive outcomes associated with this regime [32]. Although this multimodal approach has proved efficient for the treatment of children with low-grade PBTs, children with high-grade PBTs, including DIPG, relapsed medulloblastoma, and ependymoma, still face extremely poor prognoses. Pediatric patients with DIPG tend to have worse prognoses: 70–90% of these patients die within 2 years of diagnosis, due to the inability to carry out surgical resection and unresponsiveness to radiation therapy alone or in combination with chemotherapy [33,34,35]. Notably, the success rate of new therapies in PBTs is the lowest among those for major pathologies [36].

### 2.2. CAR Targets

#### 2.2.1. Antigen Prerequisite for CNS Tumor CAR T Cell Targeting

One of the first considerations in engineering CAR T cells for solid tumors is the identification of candidate antigens. An ideal CAR target is one that is overexpressed on tumor cells, to maximize potency, while absent on normal tissue, to minimize toxicity. As mentioned above, PBTs harbor a small subpopulation of self-renewing cells that are considered responsible for tumor initiation, maintenance, and recurrence [27]. Thus, it is essential for the antigen target of CARs to be expressed on this subset in addition to the tumor bulk to prevent immune escape.

CAR T cell infusions in patients are often associated with cytokine release syndrome, which correlates with CAR T cell activity [37]. With regard to brain tumors, these effects can induce both severe off-tumor on-target and off-tumor off-target acute neuroinflammatory toxicities, as evidenced in small animal models [38]. It is very difficult to find a target antigen for CARs on solid tumors that is not expressed on healthy tissue. Current CAR targets correspond to tumor-associated antigens (TAAs) that are maximally expressed on tumor cells and minimally expressed on normal tissues to minimize on-target off-tumor toxicities. For example, CD19-specific CAR T cells induce B cell aplasia with subsequent hypogammaglobulinemia that is effectively manageable with immunoglobulin replacement therapy [37].

#### 2.2.2. Associated CAR Target for Pediatric CNS Tumors

The genome stability described in most PBTs often leads to a poor generation of neo-epitopes in comparison to those reported in adult cancers [39]. A pool of five tumor-associated antigens (B7-H3, GD2, ILR13α2, HER2, and EPHA2) has, nevertheless, been identified in PBTs, mostly as a result of research on adult GBM [38,40,41,42] These antigens have recently been ranked according to their abundance across a panel of 49 patient-derived orthotopic xenograft (PDOX) samples, including medulloblastomas, ATRTs, ependymomas, and high-grade gliomas (HGGs). Among these five antigens, B7-H3 and GD2 were found to be the most consistently expressed antigens (in about 83% of the analyzed samples) [41]. ILRα2 expression was evidenced in about 67% of the studied samples, followed by HER2 (36%) and EPHA2 (28%). Notably, the cell-surface expression of these antigens correlated neither with the existing gene expression (RNAseq) data for the matching PDOX samples nor the samples from the Pediatric Genome Project [41].

The potency and safety of second-generation CAR T cells specific to these targets have been evaluated in PBT PDOX models [38,40,42]. For instance, B7-H3-specific CAR T cells were found to efficiently control tumor growth in several PBT PDOX small animal models in the absence of any toxicity despite B7-H3′s expression on normal cells [40,42]. GD2-specific engineered T cells induced potent and lasting tumor clearance in a PDOX of DIPG bearing the H3K27M mutation [38]. However, an enhanced-affinity GD2-specific second-generation CAR was later reported to induce lethal central nervous toxicity, comprising extensive CAR T cell infiltration and proliferation within the brain as well as neuronal destruction [43]. These results have been contested but have been reproduced more recently by others [44,45]. In this regard, it should be kept in mind that GD2-specific therapeutic antibody infusions are frequently associated with severe neurotoxicity due to GD2 expression in peripheral nerves and the brain parenchyma [46,47]. The same safety concern applies to HER2. While potent antimedulloblastoma and safe activity of HER2-specific CAR T cells has been reported in small animal models of medulloblastomas, safety considerations regarding HER2 remain critical, with the death of the first patient treated with HER2-specific CAR T having been reported [48,49]. Notably, the HER2-specific antibody trastuzumab has known on-target off-tumor toxicities [50]. The epithelial cell receptor protein kinase EPHA2 has been found to be particularly overexpressed on group 3 medulloblastomas and PFA ependymomas [51]. EPHA2-specific CAR T cell therapy has been proven to be effective and safe in PDOX models of medulloblastoma and ependymomas, even though it is also detected at low levels on epithelial cells as well as on brain cells [52].

**Figure 1:**
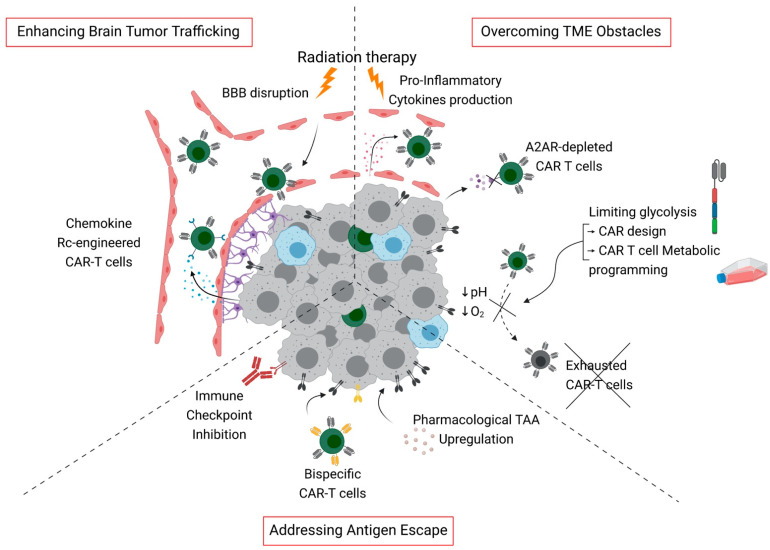
Strategies for improving the efficacy of CAR T cell therapy in pediatric brain tumors. Top left: CAR T cell trafficking into the tumor site depends on the expression of receptors for chemokines secreted by the tumor. CAR T cells endogenously express chemokine receptors, but their respective ligands are not expressed by tumor cells. CAR T cells can be engineered to express receptors (e.g., CCR4) for chemokines secreted by tumor cells to improve CAR T cell homing to tumors. Alternatively, CAR T cell infusion can be combined with radiation therapy, which induces the release of proinflammatory cytokines to increase CAR T cell recruitment into the tumor. Bottom: Tumor cells escape CAR T cell killing by downregulating CAR target expression. CAR target expression can be induced using epigenetic molecules. Bi- or multispecific CAR T cells can be used to avoid the emergence of antigen-negative tumor clones. Antigen-activated CAR T cells in the TME upregulate immune checkpoint molecules that induce T cell dysfunction and tumor escape. ICM inhibitors can be used as adjunct therapy, or CAR T cells can be genetically modified to avoid the expression of ICM receptors and T cell dysfunction. Top right: The hypoxic tumor microenvironment is rich in soluble factors such as immunosuppressive cytokines and tumor metabolites that can inhibit CAR T cells directly or indirectly. A2AR genetic depletion in CAR T cells increases T cell resistance to adenosine. Glutaminolysis inhibitors, CAR design, and/or T cell engineering impact CAR T cell fitness and antitumor efficiency. CAR T cells can be further engineered to express activating receptors specific for immunosuppressing cytokines present in the TME. Lastly, adjunct radiotherapy can be used to tame the immunosuppressive TME and increase CAR T cell antitumor activity.

Based on the above promising proof-of-concept studies, clinical trials for PBTs have recently opened (Table 2). Notably, the clinical trial experiences with IL-13R2α-, EGFRvIII-, and HER2 CAR T cells in adult patients provided the first evidence of the safety of this approach. However, only a few patients appeared to show benefits from CAR T infusions, emphasizing the important challenges facing CAR T cell therapy for PBTs. The key findings from these three clinical studies were (i) the variable expansion and trafficking of CAR T cells to the brain, (ii) the dynamic immunosuppressive response raised by the TME, and (iii) antigen loss in post-therapy recurrent tumors [17,53,54]. We will discuss each of these challenges and review the strategies for addressing them in the context of PBTs in the sections below (Figure 1).

#### 2.2.3. Addressing Antigen Escape

The identification of novel tumor targets for PBTs will provide new options for CAR T cell development. In particular, the capacity of GSC targeting will, likewise, decrease tumor recurrence. Novel antigens can be found using proteomic, DNA, or RNA sequencing analysis in comparison with human single-cell atlases [14]. As recurrent tumors will still, however, be able to escape monospecific CAR T cell therapy through the emergence of antigen-negative clones, combinatorial antigen recognition strategies have been developed. For the last, targets are often selected from a collection of well-characterized tumor antigens for safety concerns. Bispecific CAR molecules have been designed by incorporating two scFvs, one for HER2 and the second for IL13Rα2, joined in tandem for the therapy of adult GBM [55]. The bispecific HER2/ILR13Rα2 tandem CAR T cells could mitigate antigen escape and improve survival in a GBM PDOX model compared to unispecific CAR T cells, a pool of thereof and a product of T cells co-expressing both CARs [55]. This approach was furthermore extended with a third EPHA2-specific scFv domain to obtain a trispecific CAR [56]. However, late tumor relapse has been evidenced in all the research, with the loss of all the targeted antigens [55,56]. In addition, it remains unclear how the toxicity profile of tandem CAR T cells compares clinically to that of unispecific CAR T cells, though it has been suggested that bispecific tandem CAR T cells may display a greater capacity to distinguish tumor cells from normal cells. To date, no tandem CAR trials for pediatric CNS tumors have occurred. In addition, the minimum antigen density threshold required for optimal CAR T cell activation must be determined in order to anticipate antigen escape. Notably, CAR T cell recognition often necessitates a high level of expression to fully activate the T cells [57].

To address antigen expression heterogeneity, pharmacological modulation strategies have been developed. An example is the ability of epigenetic modulators to increase GD2 tumor cell-surface expression. In a preclinical model of Ewing’s sarcomas, the inhibition of enhancer of zeste homolog 2 (EZH2) selectively upregulated GD2 expression in GD2-low or GD2-negative Ewing’s sarcoma cells [57]. Similar results were obtained when employing the histone deacetylase inhibitor vorinostat in preclinical models of neuroblastoma [58,59]. In this approach, it is essential that (i) the respective modifier crosses the blood–brain barrier, and (ii) that antigen expression upregulation remains specifically limited to tumor cells to avoid on-target off-tumor toxicities. Given the broad spectrum of the biological effects of epigenetic modifiers, the design of immune combination therapies should also address their effects on the tumor cell phenotype, the CAR T cell activity, and the TME immune-suppressive components [60].

Radiotherapy represents another strategy for overcoming antigen expression heterogeneity. Beyond the secretion of cytolytic granules upon the engagement of the chimeric antigen receptor by the TAA, CAR T cells also mediate target cell apoptosis through alternative mechanisms via the engagement of the Fas/FasL or TRAIL axes [61]. Recent research suggests that these mechanisms can be induced after tumor exposure to radiotherapy [62]. In particular, De Selm et al. studied an orthotopic pancreatic tumor model that is partially antigen-negative [62]. These tumors could not be eradicated by specific CAR T cell treatment, whereas the combination of CAR T cells with low-dose radiation therapy decreased antigen-negative tumor relapse. The authors demonstrated that CAR T cells, upon expressing TRAIL, engaged antigen-positive tumor cells, which, furthermore, allowed them to eliminate antigen-negative tumor cells that were sensitized to TRAIL-mediated cell death by low-dose radiotherapy [62].

## 3. CAR T Cell Homing in the Brain Tumor Site

### 3.1. Lymphocytes’ Access to the Brain

CAR T cells targeting brain tumors can be delivered via the blood, via the cerebrospinal fluid (CFS), or locally in the tumor site. The most efficient delivery route, however, remains to be determined. Systemic delivery, whereby CAR T cells are administrated intravenously (IV), is simpler. In this case, the BBB represents an immediate obstacle to lymphocyte trafficking to the tumor site. From the circulation, lymphocytes must first adhere to the endothelial lining of a post-capillary venule. This step involves a distinct combination of leukointegrins, chemokine receptors, and their respective endothelial cell ligands, which remain, to date, largely unknown in the context of CAR T cell homing to the brain [63].

**Figure 2:**
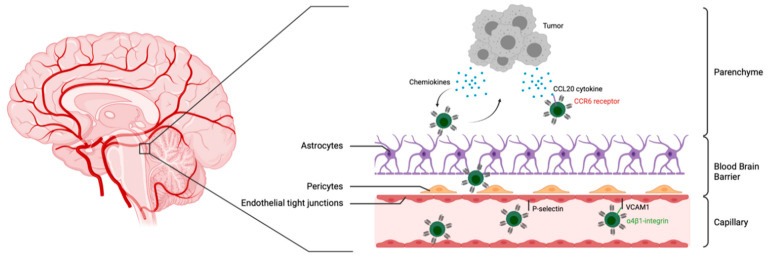
CAR T cell migration across the blood–brain barrier in pediatric brain tumors. Bottom: Tight junctions between endothelial cells limit the passage of CAR T cells. Interaction with P-selectin decreases T cell velocity in capillaries. Integrin α4β1 receptor engagement by VCAM1 proteins expressed by endothelial cells initiates T cell arrest followed by T cell diapedis. Middle: Pericytes and astrocyte endfeet further impede T cell trafficking into the brain parenchyma. Top: Tumor cells within the brain parenchyma may recruit T cells through chemokine production as indicated.

Much of what is known about T cell infiltration into the brain results from research that has been performed in disease or injury models, and has focused on CD4^+^ T cells [64,65,66]. The BBB limits and tightly controls immune cells’ entry into the brain due to the presence of an endothelial lining with tight junctions and an astrocyte foot process known as glia limitans [67]. Primed/activated T lymphocytes, not naïve or resting memory T cells, can, however, be readily recruited beyond the BBB in the absence of inflammation [64]. The initial contact with the endothelial cell lining for lymphocytes is triggered by the interaction of the activated-lymphocyte rolling receptor α4β1 integrin and its endothelial ligand VCAM1. This enables T cells to decrease their velocity and allows a lymphocyte G-protein-coupled receptor to engage an appropriate chemotactic ligand on the endothelial cells. This interaction leads to the activation of LFA-1 leukointegrin on the T cell, which subsequent binds to its endothelial CAM counter-receptors ICAM-1/-2, with firm adhesion, followed, finally, by diapedesis across the BBB. Lymphocytes must further migrate across to the glia limitans to ultimately access the brain parenchyma [67]. Subsequent migration to the tumor site is regulated by the T cell expression of chemokine receptors corresponding to the chemokine ligand secreted by tumor cells [68] (Figure 2).

Lymphocytes can also enter the CNS parenchyma through the choroid plexus located in the cerebral ventricles of the brain [69]. To access the cerebrospinal-fluid-filled ventricles, lymphocytes extravasate across fenestrated capillaries to reach the choroïd plexus parenchyma before breaching the brain cerebrospinal barrier (CSFB) [69]. The implication of P-selectin, the CCR6 receptor, and its ligand, CCL20, seems relevant in this pathway [70]. Further research has evidenced that primed Th1 cells injected intracerebroventricularly (ICV) cross the ependymal layer of the ventricle and migrate within the brain parenchyma in a chemokine-signaling-dependent fashion [71]. Therefore, this route of administration can also be used to bypass the BBB and the glia limitans.

Notably, brain tumors regularly disrupt the BBB’s integrity to an extent that differs among patients and within tumors as suggested by imaging studies [72]. Moreover, brain tumor cell infiltration extends well beyond the radiological limits of the tumor into the peritumoral tissue where the BBB remains intact and, importantly, where tumor recurrence generally occurs [73,74]. Indeed, CAR T cells can be delivered directly into the tumor site or directly into the margin of the surgical resection corresponding to the peritumoral brain tissue. One potential limitation is that site-specific administration is often more challenging than intravenous administration.

### 3.2. Route of Administration for CAR T Cells for Brain Tumor Therapy

Research using PDOX models of PBT has indicated, however, that CAR T cell trafficking into the tumor site is not affected by the route of administration [40]. Systemic CAR T cell delivery in mice resulted in therapeutic T cell trafficking into the tumor site [38,42]. However, locoregional delivery, i.e., ICV or intratumoral (IT), provides greater antitumor activity and lower systemic proinflammatory cytokine production in these models [40].

Information on the CNS penetration of CAR T cells in children with brain tumors remains absent. The scarce data available from clinical studies in adult patients with GBM have evidenced a selective trafficking of EGFRvIII-specific CAR T cells after systemic delivery, even though antitumor activity was limited [54]. Compared to preinfusion tumor biopsies, the postinfusion T cell infiltrate appeared to be enriched with EGFRvIII-specific CAR CD8^+^ T cells featuring an activated phenotype [54]. A transient complete response was also observed in one patient with multifocal GBM after multiple ICV and IT administrations of IL-13Rα2-specific CAR T cells [17]. In this patient, intracavity therapy appeared to control local recurrence, but the progression of GBM was later observed at a distant site [17]. This suggests that locoregional infusion does not necessarily translate into effective delivery to the tumor site, in contrast to preclinical observations obtained in small animal models [40]. In fact, the translation of data from rodents to humans involves the consideration of several characteristics of rodent and human brain anatomy. While similarities between the BBB and capillary characteristics among rodents and humans exist, the trafficking distances are much longer in humans, due to the size and volume of the CSF spaces.

### 3.3. Strategies for Enhanced Brain Tumor Trafficking

In addition to the adhesion molecules discussed above, multiple chemokines regulate leukocyte homing. For instance, CXCR3 and its ligands CXCL9 and CXCL10 are known to play a key role in recruiting effector Th1 and CD8^+^ lymphocytes into the TME [75]. These fundamentals apply evenly to CAR T cells, which must express the respective chemokine receptor for the specific tumor-secreted chemokine ligand to localize successfully to the tumor site [76]. However, tumors often express specific chemokines that deregulate the immune response. For instance, a high concentration of CXCL12 in the TME induces CXCR4 downregulation in T cells, which prevents CD8^+^ T cell tumor infiltration [77]. In adult GBM, CCR4 chemokine ligands are often upregulated, which supports CCR4^+^ regulatory T cell homing in the TME [78].

To improve CAR T cell migration to the tumor, a chemokine-receptor-engineered T cell can be designed to match the specific chemokine signaling of a tumor. For example, the forced expression of CCR4, which is absent on effector CD8^+^ T cells, increases both CAR T cell tumor infiltration and responses in small animal models with Hodgkin lymphoma [79]. CCR4 could, furthermore, be a strategy adaptable to the context of PBTs, as it seems to play a critical role in tumor progression [80,81,82]. Additionally, the CXCL5/CXL6 chemokine receptor could represent an important strategy for increased CAR T cell trafficking in ATRTs [83].

Lastly, studies suggest that radiotherapy could be used to increase the BBB permeability at the tumor site. For example, the BBB in DIPGs remains intact, based on the observation that these tumors are not significantly enhanced after contrast agent injection. However, increased enhancement, which is probably associated with BBB disruption, is often observed after radiotherapy. This could represent a window for CAR T cell delivery in this tumor group, as suggested in a recent preclinical study indicating that radiation therapy promotes the rapid extravasation of CAR T cells through the BBB and expansion in the TME [84]. Further work is needed to understand the molecular mechanisms involved in the homing of CAR T cells to the brain and to determine if the delivery route affects safety and/or patient outcomes.

## 4. The Antagonistic Pediatric Brain Tumor Microenvironment

### 4.1. Subgroup-Specific Immune Microenvironment in Pediatric Brain Tumors

Because the response to CAR T cell therapy in patients is tightly correlated to the cells’ activity and persistence, the understanding of the immune microenvironment composition of PBTs is key for effective treatment. With recent technological developments, TME immunoprofiling has evolved from examining one marker by immunochemistry (IHC) to considering multiple subsets and phenotypes by multiplex IHC, high-dimensional mass and/or flow cytometry, and single-cell RNA sequencing. The application and integration of these multiplex techniques have been, however, limited in pediatric neuro-oncology, mostly because of the relatively small disease population. Overall, current research indicates that most PBTs trend toward an “immune cold” TME, with a low mutational burden, low T cell infiltration, and high myeloid signatures [85,86].

In the glial group, a thorough analysis of transcriptional signatures distinguishes three distinct glial tumor subtypes that are more inflamed than others are. The “immune hot” subtype, having the highest fractions of tumor-infiltrating immune cells, is followed by an intermediate inflamed “immune altered” subtype and, lastly, an “immune cold” tumor subtype, which features the lowest tumor-infiltrating T cell fraction. “Immune hot” glial tumors express the highest levels of PD-L1 [85]. Notably, infused CAR T cells express PD-1 and, therefore, are susceptible to PD-L1-mediated suppression [87]. Thus, blocking PD-1 could synergize with CAR T cell therapy in improving outcomes in children with PD-L1-expressing PBT. Engineered CAR T cells that secrete blocking antibodies against PD-1/PD-L1 or the genetic removal of PD-1 from CAR T cell products by CRISPR-Cas9 gene editing have resulted in improved effector functions in small animal models and, thus, can be proposed as alternative approaches [88,89].

Most medulloblastomas also fall into the “immune cold” tumor subtype [90]. However, relatively high PD-L1 expression is found in the WNT and the SHH molecular subgroups compared to group 3 and group 4 [90]. In addition to immune checkpoints, immunosuppressive cytokines seem to provide another inhibitory signal depending on the molecular subgroup [90]. This immunosuppressive cytokine signaling can be overturned by expressing a cytokine switch receptor (CSR), which turns an immunosuppressive signal into an activating signal. Examples of CSRs include TGF-β CSR consisting of a TGF-β-specific scFv and an activating CD28 signaling domain [91]. Activating cytokine receptors can also be constitutively triggered without the need for cytokine binding, thus providing CAR T cells with a survival signal in the absence of cytokine production in the TME. In this regard, Shum et al. modified CAR T cells with a constitutively signaling IL-7 cytokine receptor (CR7), which lacked the IL-7 extracellular binding domain, to promote the persistence and antitumor activity of second-generation GD2-specific CAR T cells against neuroblastoma. Similar results were obtained in this study in an orthotopic GBM mouse model with EPHA2-specific CAR T cells co-expressing CR7 [92]. Importantly, this proof-of-concept investigation further led to the opening of a phase I clinical trial for evaluating the safety of GD2-specific CAR T cells expressing CR7 in children with DIPG (ClinicalTrials.gov Identifier: NCT04099799, Table 2).

The same strategies could be considered for ependymomas, which have significantly more infiltrating myeloid cells and T lymphocytes than medulloblastomas or high-grade gliomas [86]. In particular, the supratentorial RELA fusion ependymoma subtype expresses a higher PD-L1 level than other ependymomas [93].

### 4.2. Metabolic Barriers

Most cancer cells engage in aerobic glycolysis, even in the presence of oxygen, to satisfy their tremendous anabolic and energetic requirements for multiplication and survival [94]. In addition, tumor cells commit glutaminolysis to produce intermediate metabolites for lipid, protein, and nucleic acid synthesis [95]. Notably, activated T cells operate these same metabolic programs to sustain cell proliferation during clonal expansion [96]. CAR T cells, as with other effector T cells, require the same specific metabolic support for optimal proliferation and effector functions [97]. As a result, not only do tumor cells and tumor-infiltrating T cells compete for limited nutrients, but specific metabolites present in the TME (discussed below) can suppress antitumor immunity. As such, effector CD8^+^ T cells often lose their proliferation capacity and effector function before achieving sufficient tumor cell elimination in the nutrient- and oxygen-deprived TME [28]. Recent studies have indicated that this process can be prevented by different means [97]. Studies show that limiting glycolysis and promoting mitochondrial metabolism during priming allow more CD8^+^ T cells to enter the memory T cell pool, which possesses superior antitumor function and persistence after administration in vivo [98]. In particular, the CAR T cell manufacturing process offers an opportunity for ex vivo metabolic reprogramming. First, the CAR design itself can reprogram the cell metabolism. For instance, compared to the CD28 signaling domain, the 4-1BB signaling domain increases mitochondrial biogenesis and oxidative phosphorylation upon activation, allowing the production of memory T cells with a better in vivo persistence phenotype [99]. The composition of the culture medium in which CAR T cells are expanded ex vivo is critical for adjusting the specific metabolic program and increasing memory cell generation. For example, the presence of a glycolysis inhibitor can enhance the generation of T cells with a memory phenotype [98]. Similar results can be obtained using lactate dehydrogenase, AKT, or glutaminolysis inhibitors during the ex vivo expansion of T cells [100,101,102].

### 4.3. Tumor-Derived Immunosuppressive Factors

Using an integrated approach incorporating proteomics, phosphoproteomics, whole-genome sequencing, and RNA sequencing, Petralia et al. have evidenced a subset of HGG that has significant upregulation of adenosine producers [29]. As a soluble metabolite, adenosine plays an important role in establishing the immune-suppressive TME [103]. In particular, adenosine restricts the proliferative and cytotoxic potential of T lymphocytes through the high-affinity adenosine receptor A2A (A2AR), and the suppressive effects of adenosine on CAR T cells were recently confirmed as antigen-specific activation-induced upregulation of A2AR expression on CAR T cells [104]. Consequently, A2AR-depleted CAR T cells showed better resistance to exhaustion, associated with a low PD-1 expression level and superior antitumor activity in vivo [104]. The CAR T cell therapy of HGG could also be potentiated by the pharmacological inhibition of A2AR2 [105].

Petralia et al. have furthermore evidenced a unique cluster in the HGG group characterized by the upregulation of glutamate receptor signaling and neurotransmitter transport pathways associated with the decreased gene expression of both CD4^+^- and CD8^+^-lymphocyte-related genes [29]. The authors suggested that a glutamate-/glutamate-receptor-mediated mechanism of tumor progression might occur in this HGG tumor subset [29]. Notably, other investigators have focused on manipulating glutamine metabolism in the TME, and recent research highlights that glutamine inhibitors and the transient inhibition of glutaminase increase T cell effector functions [106]. Thus, targeting glutamine metabolism may represent a means of enhancing CAR T cell therapy in this HGG tumor cluster.

Lastly, combination radiation and CAR T cell therapy can enhance efficacy over either modality alone. Radiotherapy can modulate the TME in a dose-dependent fashion through the induction of proinflammatory chemotactic factors or the enhancement of CAR target tumor expression, as evidenced in small animal models [70,107]. Thus, radiotherapy may help to address the shortcomings of CAR T cells in brain tumors, as discussed above [84]. However, radiation-induced inflammation often leads to a wound-healing immunosuppressive TME that is characterized by notably increased TGF-β secretion [108]. In this regard, CAR T cells expressing TGF-β cytokine switch receptors could be used in combination with radiation therapy. Another concern with regard to hypofractionated radiation therapy is the depletion of T cells in the irradiated field, because this is expected to attenuate CAR T cell persistence [109]. Thus, there is a need for continued research on the topic as a combination with CAR T cell therapy to determine the best sequence of radiation and CAR T cell therapy.

## 5. Conclusions

CAR T cell therapy represents a very attractive approach in the field of pediatric neuro-oncology for overcoming resistance to classical treatment. While promising preclinical studies have already led to the implementation of several clinical trials in children with brain tumors, the current clinical data indicate that the efficacy of CAR T cells used as a monotherapy is not especially effective in solid tumors. While clinical activity is generally achieved without on-target off-tumor toxicity, one should bear in mind that CAR T cell therapy is associated with serious life-threatening adverse events that require specific management. 

Adequate T cell migration and tumor infiltration, neuroinflammation resulting from CAR T cell activation, antigen escape, and CAR T cell persistence at the tumor site still represent major challenges to be overcome. The field of CAR T cells, however, continues to evolve to address these obstacles, and we believe that they are undoubtedly going to result in the development of effective and safe CAR T cell products in the future.

## Figures and Tables

**Table 1:** WHO-recognized grade 3 and grade 4 primary pediatric brain tumors.

Gliomas, Grade 4
Around 50% of pediatric tumors
Pediatric-type diffuse high-grade gliomas
Diffuse midline glioma, H3K27-altered
Diffuse hemispheric glioma, H3 G34 mutant
Diffuse pediatric-type high-grade glioma, H3 wildtype and IDH wildtype
Infant-type hemispheric glioma
**Embryonal Tumors, Grade 4**
Around 20% of CNS pediatric tumors
Medulloblastomas, molecularly defined
Medulloblastoma, WNT-activated
Medulloblastoma, SHH-activated and TP53 wildtype
Medulloblastoma, SHH-activated and TP53 mutant
Medulloblastoma, non-WNT/non-SHH
Medulloblastomas, histologically defined
Atypical teratoid/rhabdoid tumor
**Ependymal Tumors, Grade 2, 3**
Around 10% of CNS pediatric tumors
Supratentorial ependymoma
Supratentorial ependymoma, ZFTA-fusion-positive
Posterior fossa ependymoma
Posterior fossa ependymoma, group PFA
Posterior fossa ependymoma, group PFB
Spinal ependymoma
Spinal ependymoma, MYCN-amplified

**Table 2:** Ongoing clinical trials with CAR T cell therapy for pediatric brain tumors ^1^.

Clinical Trial Identifier	Phase	Target	Delivery *^2^*	Disease	Age	Status
NCT03500991	I	HER2	IT, IC	ATRT, ependymoma, GBM, and medulloblastoma	One year to twenty-six years	Recruiting
NCT04185038	I	B7H3	IT, IC	ATRT, DMG, DIPG, and medulloblastoma	One year to twenty-six years	Recruiting
NCT04661384	I	IL13Rα2	ICV	Ependymoma, GBM, and medulloblastoma	Eighteen years and older	Recruiting
NCT04196413	I	GD2	IV	DMG H3K27M mutant DIPG H3K27M mutant	Two years to thirty years	Recruiting
NCT03638167	I	EGFR	IT, IC	ATRT Ependymoma GBM Medulloblastoma	Fifteen years to twenty-six years	Recruiting
NCT04099797	I	GD2-CR7	IV	DIPG HGG	One year to eighteen years	Recruiting
NCT02208362	I	IL13Rα2	IT, IC	GBM	Twelve years to seventy-five years	Recruiting
NCT04510051	I	IL13Ra2	ICV	Brain neoplasms	Four years to twenty-five years	Recruiting
NCT04903080	I	HER2	IV	Refractory or recurrent ependymoma	One year to twenty-two years	Not yet recruiting

^1^ Table data accrued March 30, 2021; ^2^ IT, intratumoral; ICV, intracerebroventricular; and IV, intravenous.
